# Podoplanin: emerging functions in development, the immune system, and cancer

**DOI:** 10.3389/fimmu.2012.00283

**Published:** 2012-09-12

**Authors:** Jillian L. Astarita, Sophie E. Acton, Shannon J. Turley

**Affiliations:** ^1^ Department of Cancer Immunology and AIDS, Dana Farber Cancer InstituteBoston, MA, USA; ^2^ Division of Medical Sciences, Harvard Medical SchoolBoston, MA, USA; ^3^ Department of Cell and Developmental Biology, University College LondonLondon, UK; ^4^ Department of Microbiology and Immunobiology, Harvard Medical SchoolBoston, MA, USA

**Keywords:** podoplanin, CLEC-2, lymph node stromal cells, lymphatic endothelial cells, platelets, cancer-associated fibroblasts

## Abstract

Podoplanin (PDPN) is a well-conserved, mucin-type transmembrane protein expressed in multiple tissues during ontogeny and in adult animals, including the brain, heart, kidney, lungs, osteoblasts, and lymphoid organs. Studies of PDPN-deficient mice have demonstrated that this molecule plays a critical role in development of the heart, lungs, and lymphatic system. PDPN is widely used as a marker for lymphatic endothelial cells and fibroblastic reticular cells of lymphoid organs and for lymphatics in the skin and tumor microenvironment. Much of the mechanistic insight into PDPN biology has been gleaned from studies of tumor cells; tumor cells often upregulate PDPN as they undergo epithelial-mesenchymal transition and this upregulation is correlated with increased motility and metastasis. The physiological role of PDPN that has been most studied is its ability to aggregate and activate CLEC-2-expressing platelets, as PDPN is the only known endogenous ligand for CLEC-2. However, more recent studies have revealed that PDPN also plays crucial roles in the biology of immune cells, including T cells and dendritic cells. This review will provide a comprehensive overview of the diverse roles of PDPN in development, immunology, and cancer.

## INTRODUCTION

Podoplanin (PDPN) is a 36- to 43-kDa mucin-type transmembrane protein. It has homologues in humans, mice, rats, dogs, and hamsters and is relatively well conserved between species. PDPN has a wide variety of functions including regulation of organ development, cell motility, and tumorigenesis and metastasis (Wicki and Christofori, 2007; Suzuki-Inoue et al., 2011). PDPN has been identified and studied in many different contexts; thus, it has been given several names. PDPN was first described on lymphatic endothelial cells (LECs) as the E11 antigen (Wetterwald et al., 1996) and on fibroblastic reticular cells (FRCs) of lymphoid organs and thymic epithelial cells as gp38 (Farr et al., 1992a,b; **Table 1**). PDPN is also homologous to T1a/rTI_40_, one of the first molecular markers of alveolar type I epithelial cells (Rishi et al., 1995; Williams et al., 1996; **Table 1**), PA2.26, which is upregulated in skin keratinocytes upon injury (Scholl et al., 1999), OTS-8, a molecule induced in osteoblasts upon phorbol ester treatment (Nose et al., 1990), and Aggrus, a platelet-aggregating factor (Kato et al., 2003). Finally, this molecule was given the name podoplanin due to its expression on kidney podocytes and possible involvement in the flattening of podocyte foot processes (Breiteneder-Geleff et al., 1997).

**Table 1:** Podoplanin expression and function in organs and immune cells.

Organ	Time of expression	PDPN function	Reference
Central nervous system	Beginning day E9, becomes restricted to choroid plexus in adult mouse	No specific function reported during development; high PDPN expression in brain tumors	Williams et al. (1996),
			Kaji et al. (2012),
			Peterziel et al. (2012)
Heart	Expressed in entire organ on day E9; continued expression in adult heart	Required for normal heart development, specifically for EMT in epicardium-derived cells	Martín-Villar et al. (2005),
			Mahtab et al. (2009, 2008),
			Douglas et al. (2009)
Lungs	Appears in foregut on day E9 before lung buds; subsequently restricted to alveolar type I epithelial cells	Required for lung development; specifically the effective maturation of alveolar type I epithelial cells	Ramirez et al. (2003)
Intestine	Expressed on day E9 in foregut; continued expression in lamina propia	No specific function determined	Farr et al. (1992a),
			Williams et al. (1996)
Lymphoid organs	Present in spleen 4 days postnatally; in adult, expression by FRCs, LECs, and FDCs in lymph node and spleen, and thymic medullary epithelial cells	Required for proper formation and organization of lymph nodes and spleen; necessary for efficient DC migration to and within lymph nodes; highly expressed by stroma and some T cells in ectopic lymphoid tissue	Farr et al. (1992a),
			Bekiaris et al. (2007),
			Raica et al. (2010),
			Peters et al. (2011),
			Acton et al. (2012),
			Yu et al., (2007)
**Immune cell**	**Expression pattern**	**Function**	**Reference**
T cell	Expressed only on T_H_17 cells, not other subsets	Plays a role in T_H_17-driven development of ectopic germinal centers in EAE	Peters et al. (2011)
Macrophages	Expressed by inflammatory macrophages, such as thioglycollate-elicited peritoneal macrophages	Possibly plays a role in response to fungal infections; can activate platelet aggregation	Hou et al. (2010), Kerrigan et al. (2012)

While PDPN expression patterns in many of these cells have been well characterized, there is still little known about the physiological functions of this protein. PDPN has been reported to bind to the C-type lectin receptor CLEC-2, which is highly expressed by platelets and immune cells. However, this interaction has only been extensively studied with regard to platelets. Engagement of PDPN by CLEC-2 on platelets leads to platelet aggregation and activation, and this process is critical for the maintenance of normal lymphatic vessels (Bertozzi et al., 2010; Suzuki-Inoue et al., 2010). PDPN has also been used as a marker for FRCs in the lymph node (LN) and spleen, but there is limited data on whether PDPN expression is required for the function of these cells or influences their interactions with leukocytes.

The majority of data examining the function and signaling pathways of PDPN are from studies of PDPN overexpression in tumor cells. While these studies certainly provide critical insight into cellular and molecular aspects of PDPN biology, it is important to understand whether PDPN functions similarly in non-pathological settings and in cell types where it is naturally expressed. Here, we will review what is currently known about the structure, molecular interactions, and *in vivo* roles of PDPN. We will focus on the function of PDPN on stromal cells, including epithelial cells, endothelial cells, and fibroblasts but will also describe recent studies of PDPN expression by immune cells.

## PDPN IN DEVELOPMENT

Podoplanin is first expressed in the developing mouse embryo on day E9 in the foregut, proepicardial organ, and central nervous system (CNS; Williams et al., 1996; Mahtab et al., 2009; **Table 1**). Throughout development, it is also expressed in the fetal rat kidney, choroid plexus, intestine, and esophagus (Williams et al., 1996; **Table 1**). Over time, PDPN expression is increasingly restricted such that in an adult animal, PDPN is predominantly expressed in alveolar type I cells, mature osteoblasts, LECs, and FRCs in the T cell zone of lymphoid organs (Wetterwald et al., 1996; Williams et al., 1996; Schacht et al., 2003; **Table 1**). PDPN is critical for normal development of some of these organs and has been well studied in PDPN-deficient animals. *Pdpn*^-/-^ mice develop normally until around day E10, which coincides with the appearance of PDPN protein. From days E10–16, approximately 40% of *Pdpn*^-/-^ embryos die; the ones that survive to birth die within a few days (Mahtab et al., 2008). However, interestingly, when the mice are crossed onto a C57Bl/6 background, many more embryos survive to birth, and although 50% die in the first week, approximately 20% of the mice do survive to adulthood (Uhrin et al., 2010). The reason why the genetic background affects the severity of the defects suffered by the *Pdpn*^-/-^ mice is intriguing and warrants further study. Furthermore, it would be of great use to the field to have a conditional knockout of PDPN to avoid these survival defects.

The defect in blood-lymphatic vascular separation is the phenotype most extensively studied in PDPN-deficient mice. On day E11.5, PDPN first appears in the developing circulatory system on Prox-1^+^ lymphatic cells (Schacht et al., 2003). It was first reported by Schacht et al. (2003) that *Pdpn*^-/-^ mice have abnormal lymphatic vessels that cannot properly regulate lymph flow and that this defect did not appear in blood vessels. These findings were further supported by Fu et al. (2008), who reported that endothelial cell expression of PDPN was responsible for a blood-lymphatic misconnection. Furthermore, continued expression of PDPN into adulthood was required to maintain proper vascular architecture, as an inducible deletion of T-synthase, a major glycosyltransferase required for O-glycan synthesis and normal levels of PDPN expression, showed similar blood-lymph mixing (Fu et al., 2008).

This non-separation phenotype is also observed in mice where hematopoietic cells lack Syk, SLP-76, PLCγ2, and CLEC-2 (Abtahian et al., 2003; Sebzda et al., 2006; Suzuki-Inoue et al., 2010). While platelets and neutrophils both express CLEC-2, it was initially believed that platelets could not be involved in this phenotype because mice lacking nearly all platelets had normal lymphatic vasculature (Shivdasani et al., 1995). However, elegant recent studies have proven that CLEC-2 expression and downstream signaling through SLP-76 are required specifically in platelets (Bertozzi et al., 2010; Osada et al., 2012). The interaction of platelet CLEC-2 and PDPN on LECs induces platelet aggregation and prevents blood from flowing into new lymphatic vessels budding from the cardinal vein. Furthermore, injecting a PDPN-blocking antibody or otherwise inhibiting platelet aggregation is sufficient to disrupt lymphatic development (Uhrin et al., 2010). Overall, the model that has emerged indicates that during the budding of the lymph sac from the cardinal vein, PDPN becomes upregulated on Prox-1^+^Lyve-1^+^ LECs and binds with CLEC-2 on platelets. This interaction activates downstream signaling in platelets, which results in platelet aggregation. This aggregation then allows for a complete separation of the budding lymphatic vessels from the developing blood vessels.

In addition to its role in lymphatic vessel development, PDPN may play a role in the development or maintenance of lymphoid organ architecture. In the spleens of mice lacking lymphocytes, no PDPN expression is observed, although FRCs are still present as indicated by VCAM-1 and ER-TR7 staining (Ngo et al., 2001; Bekiaris et al., 2007). It appears that this lack of expression is due to a lack of lymphotoxin, but it remains unclear exactly which cell type provides that signal during development of the spleen. A more striking phenotype has been observed by Peters et al. (2011) in that *Pdpn*^-/-^ mice lack nearly all LNs, and the ones that develop are extremely disorganized. The spleens of these mice were present, but were also disorganized. It is interesting to speculate whether this phenotype indicates an important function for PDPN on FRCs and T cells; however, it is also possible that the lack of LNs is due to impaired lymph flow caused by the malformed lymphatic vessels. Thus, further work is needed to dissect this phenotype.

The first defects described in *Pdpn*^-/-^ mice were in the lung (**Table 1**), as these mice die shortly after birth due to an inability to inflate the lungs (Ramirez et al., 2003). This defect stems from an impairment in the development of alveolar type I cells. These cells cover the majority of the lung surface and play a key role in the proper development of the alveoli, which are the major gas exchange centers of the lung (Williams, 2003). In normal lung development, alveolar type I cells exhibit a high proliferation rate during early and mid-gestation periods, but this high growth rate slows a few days before birth (Ramirez et al., 2003). However, when alveolar type I cells lack PDPN, they continue proliferating in later stages of embryonic development, which is partially explained by a decrease in the negative cell cycle regulator, p21, at birth (Millien et al., 2006).

Podoplanin is also necessary for proper development of the heart (**Table 1**). PDPN is first expressed in the proepicardial organ on day E9.5 and by day E12.5, it is expressed in most of the heart. Without PDPN expression, the hearts of developing mice exhibit hypoplasia in the pulmonary vein, left atrium dorsal wall, and the atrial septum (Douglas et al., 2009). In this setting, the lack of PDPN leads to a dysregulation of epithelial-mesenchymal transition (EMT), a process that involves the transition of sessile epithelial cells into more motile mesenchymal cells through the downregulation of epithelial markers, such as adhesion molecules like E-cadherin (Thiery, 2002). In PDPN-deficient mice, the epicardium-derived cells responsible for cardiac development show increased levels of E-cadherin and decreased levels of RhoA compared with their WT counterparts, which is indicative of impaired EMT (Mahtab et al., 2008, 2009). While PDPN has been shown to play a role in regulating EMT (Martín-Villar et al., 2006), these studies are the first evidence that PDPN may play a role in physiological instances of EMT in non-transformed cells.

Overall, PDPN is crucial for the development of multiple organs, including the lymphatic system, lungs, and heart. Interestingly, PDPN serves diverse functions in these organs. In some instances it is required for CLEC-2-dependent platelet aggregation, but in others it seems to have an intrinsic effect on proliferation or differentiation in a specific cell type. This raises the question of whether PDPN function could to some degree be tissue specific. The range of physiological effects downstream of PDPN expression may be due to different protein interactions and binding partners in diverse cell types.

## MOLECULAR INTERACTIONS AND SIGNALING OF PDPN

**FIGURE 1:**
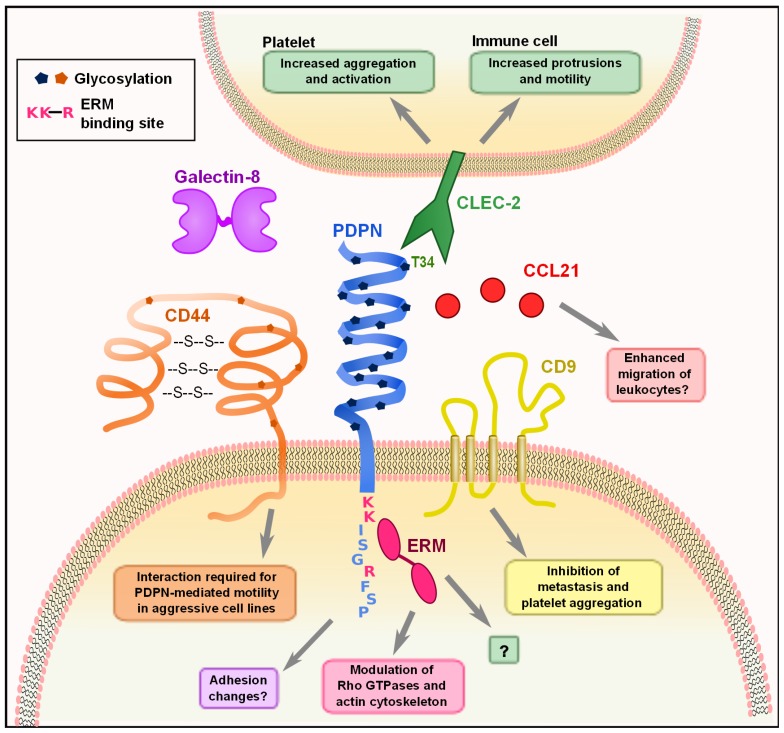
Molecular interactions of PDPN. PDPN interacts with a variety of intracellular and transmembrane proteins to mediate effects on cell migration and adhesion. The binding of PDPN to CD44 or ERMs results in increased cell migration and rearrangement of the actin cytoskeleton to generate actin-rich protrusions of the membrane. The three amino acids colored in pink (K, K, R) are the basic residues requires for ERM protein binding. Interactions between PDPN and CD9 affect metastasis and platelet aggregation. The engagement of PDPN by CLEC-2 causes increased motility in DCs and aggregation and activation of platelets. PDPN binds with high affinity to the chemokine CCL21 and while the consequences of this effect have not been examined, it may play a role in facilitating leukocyte migration. Finally, PDPN binding to galectin-8 may modulate adhesion of LECs.

Podoplanin contains a single transmembrane domain, a short, nine amino acid cytoplasmic tail, and a heavily glycosylated extracellular domain (Martín-Villar et al., 2005). While there are no obvious conserved protein domains in PDPN, several studies have identified specific residues on PDPN that mediate interactions with other proteins (**Figure 1**). The first hints at the cellular function of PDPN came from Scholl et al. (1999), who discovered that PDPN was upregulated in keratinocytes from induced epidermal carcinogenesis and was localized to membrane protrusions such as filopodia and lamellipodia. PDPN co-localized with ezrin, radixin, and moesin (ERM) family proteins, and was later found to directly bind ezrin and moesin. This interaction requires a conserved motif of three basic residues in the cytoplasmic tail (see **Figure 1**) and overexpression of PDPN resulted in increased phosphorylation of ERM proteins (Martín-Villar et al., 2006; Wicki et al., 2006). The ERM proteins function as connectors between integral membrane proteins and the actin cytoskeleton. Phosphorylation causes a conformational change that exposes binding sites for actin and other proteins (Fehon et al., 2010). Thus, this interaction likely underlies many of the effects that PDPN has on cytoskeleton. A closer examination of the effects of PDPN upregulation revealed that overexpression of PDPN in epithelial cell lines caused them to become more mesenchymal in appearance, with decreased stress fibers and increased filopodia (Martín-Villar et al., 2006; Wicki et al., 2006). These changes, in addition to a downregulation of E-cadherin and other epithelial markers, are indicative of cells undergoing EMT, which is indeed what Martín-Villar et al. (2006) observed. However, Wicki et al. (2006) demonstrated that while PDPN overexpression resulted in increased motility, it did not result in an E-cadherin switch or EMT. Discrepancies were also found when the involvement of Rho family small G proteins was examined. Martín-Villar et al. (2006) reported that PDPN overexpression resulted in an increase in RhoA and no change in Rac-1 or Cdc42, while Wicki et al. (2006) found a downregulation in RhoA, Rac-1, and Cdc42. In addition, Navarro et al. (2010) found that knocking down PDPN in LECs resulted in decreased levels of activated RhoA and increased levels in Cdc42. While it is clear that the expression of PDPN has an effect on the activity levels of these proteins, more work must be done to fully elucidate the mechanism. As described above, it is possible that PDPN exerts different effects and utilizes distinct signaling cascades in various cell types, which could partially explain the observed discrepancies.

Recently, it was discovered that PDPN resides in lipid rafts in the plasma membrane. Barth et al. (2010) found that PDPN resides in detergent-insoluble fractions of alveolar type I epithelial cells, but its function within these rafts remains unknown. It was subsequently reported that human PDPN expressed in Madin–Darby Canine Kidney (MDCK) type II cells is localized to lipid rafts (Fernández-Muñoz et al, 2011). In these cells, the transmembrane and cytoplasmic domains of PDPN were necessary for association with lipid rafts. Furthermore, manipulation of this localization by substituting the transmembrane domain with that of other proteins inhibited PDPN-mediated increases in EMT, migration, and phosphorylation of ERMs (Fernández-Muñoz et al, 2011). Interestingly, cytoskeletal interactions are not required for PDPN to get into lipid rafts (Barth et al., 2010); however, the cytosolic domain is necessary (Fernández-Muñoz et al, 2011) and one way this might be explained is via interactions with ERMs, given that ezrin is also raft-associated.

Given that the cytoplasmic tail of PDPN is extremely short, it is difficult to imagine that there is much direct signaling downstream of PDPN other than through the ERM proteins, simply due to spatial restrictions. Interestingly, however, PDPN also interacts with two integral membrane proteins that could help to further explain how it affects cell motility and metastasis. CD44, which is widely expressed, affects many cellular functions such as migration and adhesion, and the expression of some isoforms is linked to more invasive cancers. Martín-Villar et al. (2010) noted that CD44 and PDPN were coordinately upregulated in aggressive cancer cell lines and subsequently found that they directly bind to one another. This interaction is dependent on correct glycosylation of the extracellular domain of PDPN, and CD44 expression is required for PDPN-induced cell migration (Martín-Villar et al., 2010). Additionally, Nakazawa et al. (2008) found that PDPN directly interacts with the tetraspanin CD9 through transmembrane domains 1 and 2 of CD9. CD9 acts as a tumor suppressor in many cancers (Zöller, 2009), and co-expression of CD9 and PDPN resulted in a CD9-mediated decrease of PDPN-induced metastasis. CD9 also inhibited PDPN-mediated platelet aggregation without directly interfering with CLEC-2 binding of PDPN (Nakazawa et al., 2008). This finding indicates that CD9 potentially disrupts CLEC-2 multimerization, which is required for downstream signaling. These interactions provide some insight into how PDPN can exert striking effects on actin cytoskeleton rearrangement, cell motility, and metastasis. Still however, much remains to be elucidated such as the downstream signaling changes that occur upon PDPN binding to CD9 or CD44, how PDPN overexpression results in an increase of ERM phosphorylation, and how that in turn modulates the activity of the Rho family small G proteins.

The only known receptor for PDPN is CLEC-2, a C-type lectin that is expressed by platelets, neutrophils, and dendritic cells (DCs) (Colonna et al., 2000; Sobanov et al., 2001; Kerrigan et al., 2009; Acton et al., 2012). Glycosylation of T34 on PDPN is required for CLEC-2 binding of PDPN. This amino acid resides in the platelet-aggregation stimulating (PLAG) domain, which is highly conserved between PDPN homologues (Kaneko et al., 2006). The effect of CLEC-2 engagement by PDPN has been extensively studied in platelets; however, the effect of this interaction in PDPN-expressing cells has not been addressed. This is an area that warrants further exploration, given that *in vivo*, many PDPN^+^ cells will be exposed to CLEC-2 signals, whether they are tumor cells interacting with CLEC-2^+^ platelets or FRCs interacting with CLEC-2^+^ DCs.

Lymphatic endothelial cells and FRCs, the two major subsets of lymphoid stromal cells, express high levels of PDPN (Malhotra et al., 2012; **Table 1**), but only a few studies have examined the molecular function of PDPN in these cells. PDPN interacts with galectin-8 on LECs, and this interaction is also dependent on PDPN glycosylation (Cueni and Detmar, 2009). Galectin-8 can have varying effects on adhesion depending on whether it is secreted or membrane-bound (Zick et al., 2004); it seems that PDPN binding to galectin-8 may affect LEC adhesion, but additional studies are needed to fully elucidate the consequences of this interaction. PDPN also binds CCL21 with high affinity, and this interaction is also dependent on glycosylation of PDPN (Kerjaschki et al., 2004). This interaction has interesting implications for lymphocyte trafficking, as both LECs and FRCs express CCL21 to direct lymphocyte and DC trafficking to the T zone of LNs (Luther et al., 2000; Bajénoff et al., 2006; Turley et al., 2010).

It has yet to be examined whether the above binding partners of PDPN are cell-type specific or how interaction with one protein affects the binding of PDPN to another interacting molecule. With the exception of the ERMs and CD44, it remains unclear whether PDPN can bind to several of these proteins at one time or whether such interactions might be mutually exclusive. A more global understanding of these various interactions is critical to our overall understanding of PDPN’s molecular functions and downstream signaling.

## TRANSCRIPTIONAL CONTROL OF PDPN EXPRESSION

Information about the transcriptional control of PDPN first came from the early studies of the role of PDPN in the development of the lymphatic system. The fact that PDPN was specifically expressed on differentiating LECs but not nearby BECs led to the discovery that Prox-1, the major regulator of LEC differentiation, controlled the induction of PDPN (Hong et al., 2002). In fact, forced expression of Prox-1 was sufficient to induce a LEC-like phenotype in differentiated BECs, including the upregulation of PDPN (Hong et al., 2002). Furthermore, it was later found that IL-3, which is involved controlling the differentiation of a variety of hematopoietic cells and is produced by LECs but not BECs, was capable of upregulating Prox-1 and PDPN (Gröger et al., 2004). However, Prox-1 is not expressed in FRCs or in many of the other cells types expressing PDPN. Therefore alternative pathways must be involved in PDPN expression in tissues other than lymphatics. This may be another reason why the physiological functions of PDPN are so varied between different systems.

**FIGURE 2:**
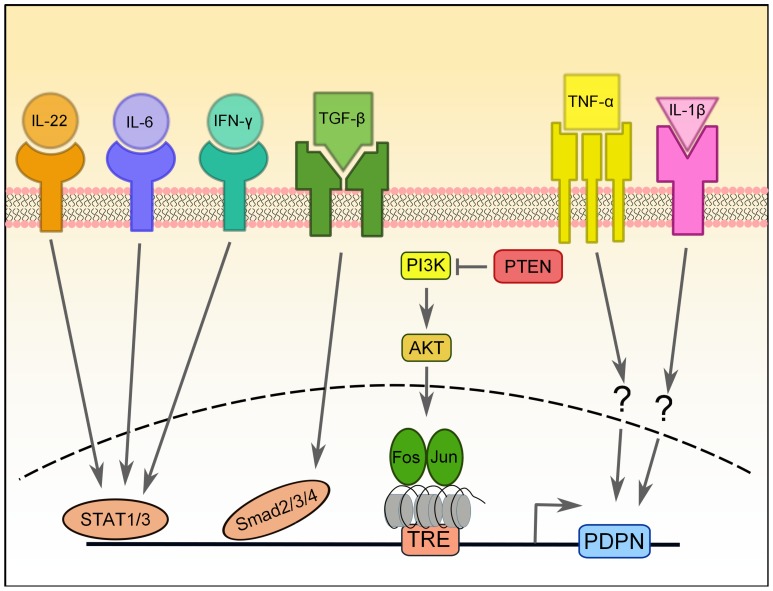
**Transcriptional regulation of PDPN expression.** PDPN expression can be upregulated by a number of pro-inflammatory cytokines, including IL-22, IL-6, IFN-γ, TGF-β, IL-1β, and TNF-α, but the signaling pathways involved are largely unknown. PDPN upregulation induced by TGF-β requires Smad2/3 and 4 activity, while upregulation induced by IFN-ψ depends on STAT1 and STAT3 and that of IL-6 and IL-22 depends on STAT3. The PI3K-AKT-AP-1 pathway can also induce PDPN expression in brain tumors that have lost the negative regulation normally provided by PTEN. AP-1, a transcription factor comprised of Fos and Jun proteins, binds to the tetradecanoylphorbol acetate-responsive element (TRE) in the promoter of PDPN, which is heavily methylated.

In skin cancers, osteosarcomas, and gliomas, PDPN is regulated by the AP-1 transcription factor (Durchdewald et al., 2008; Kunita et al., 2011; Peterziel et al., 2012). AP-1 is a heterodimeric complex comprised of Fos and Jun proteins. Both Fos and Jun are critical for progression of many carcinomas, including models of skin carcinogenesis (Eferl and Wagner, 2003). Durchdewald et al. (2008) compared genetic profiles of skin tumors from mice that had either WT Fos expression or Fos specifically deleted in keratinocytes and found that PDPN was one of the most highly upregulated genes in the Fos-sufficient samples. Furthermore, they demonstrated that Fos directly binds to the PDPN promoter. This interaction was further characterized in gliomas, and it was found that PTEN expression, a negative regulator of the PI3K-AKT-AP-1 pathway, was inversely correlated with PDPN expression (**Figure 2**; Peterziel et al., 2012). Furthermore, the PDPN promoter is heavily methylated, which keeps it repressed (Peterziel et al., 2012). Thus, it appears that a major pathway of PDPN upregulation in malignant conditions depends on the activity of Fos and Jun (AP-1) transcription factors.

Finally, there have been two reports of pro-inflammatory cytokines resulting in PDPN upregulation in disease. In rheumatoid arthritis (RA), fibroblast-like synoviocytes are the main mediators of inflammation and tissue destruction and undergo a process resembling EMT during RA progression (Huber et al., 2006). Ekwall et al. (2011) recently reported that while PDPN is absent from the synovium of healthy subjects and patients with osteoarthritis, it is highly upregulated in RA patients. Furthermore, expression of PDPN in cultured synoviocytes is increased upon treatment with IL-1β, TNF-α, or TGF-β1 (Ekwall et al., 2011). Similarly, PDPN upregulation was observed in keratinocytes treated *in vitro* with TGF-β, IL-6, IL-22, or IFN-γ (Honma et al., 2012). The TGF-β-mediated PDPN upregulation required Smad2/3 and 4 signaling, while STAT1 and STAT3 were necessary for IFN-γ signaling and STAT1 was required for IL-6 and IL-22 signaling (Honma et al., 2012).

Overall, it appears that a multitude of stimuli can drive PDPN expression, including normal differentiation factors such as Prox-1 and potentially malignant factors such as pro-tumorigenic signaling pathways and pro-inflammatory cytokines. It is possible that the different pathways controlling PDPN upregulation could result in the activation of distinct downstream signaling pathways and therefore different cellular outcomes. For instance, a tumor cell and a LEC compose two distinct environments with different signaling pathways and molecules active; upregulating PDPN in these distinct signaling milieus would likely have diverse outcomes.

## PDPN FUNCTIONS IN THE IMMUNE SYSTEM

While PDPN is a well-established marker for LECs (Wetterwald et al., 1996), FRCs (Farr et al., 1992b), and follicular dendritic cells (FDCs) (Yu et al., 2007) of lymphoid organs, until very recently, no particular function had been ascribed to PDPN in these immune cell populations. Recently, a PDPN-cre mouse was generated, which will be a useful tool in targeting PDPN-expressing stromal cells (Onder et al., 2011). Our lab recently demonstrated that PDPN on FRCs and LECs interacts with CLEC-2 on DCs to promote DC motility from peripheral sites to LNs and within the T cell zone (Acton et al., 2012). We found that murine DCs expressed CLEC-2 and that their migration to draining LNs was impaired when CLEC-2 was deleted. Conversely, siRNA knock down or genetic deletion of PDPN also resulted in impaired DC migration *in vivo* and impeded motility along the FRC network *in vitro*. Furthermore, the interaction between PDPN and CLEC-2 was sufficient to induce protrusion formation in a 3D tissue engineered model. Therefore, both CLEC-2 on DCs and PDPN on stromal cells are necessary for migratory DCs to efficiently reach LNs and initiate immune responses (Acton et al., 2012).

Podoplanin signaling has intrinsic effects on the proliferation, migration, and tube formation of LECs. Navarro et al. (2008,^2010^) demonstrated that knocking down PDPN expression *in vitro* inhibited the ability of LECs to properly polarize toward a wound and migrate to close the wound. Reduced PDPN levels also decreased capillary formation when the cells were plated in a deformable 3D matrix (Navarro et al., 2008). These effects were mediated by decreased RhoA activity and increased Cdc42 activity in cells lacking PDPN (Navarro et al., 2010). The mechanism underlying this effect was further investigated by Osada et al. (2012), who found that when LECs were incubated with WT but not CLEC-2^-^^/^^-^ platelets, the migration, proliferation, and *in vitro* tube formation of LECs was inhibited. This inhibition was mediated at least in part by BMP9 released in granules from the platelets upon contact with the LECs (Osada et al., 2012). In contrast, Bertozzi et al. (2010) found that co-culture of platelets with LECs did not affect their viability or proliferation. More work is necessary to determine whether CLEC-2 signals from platelets or other cells provide important signals to LECs *in vivo*.

In addition to its high expression on stromal cells, several recent reports have described PDPN expression on hematopoietic cells, including subsets of T cells and macrophages (Hou et al., 2010; Peters et al., 2011; Kerrigan et al., 2012). Interestingly, in these cases, as in those from cancer studies, PDPN expression is usually correlated with inflammatory or disease settings. In experimental autoimmune encephalomyelitis (EAE), ectopic germinal centers form in the CNS and are believed to accelerate inflammation and disease progression (Weyand et al., 2001). T_H_17 cells are particularly important for the formation of these ectopic germinal centers and EAE progression (Jäger et al., 2009). PDPN expression has been reported in ectopic lymphoid tissues in instances of chronic inflammation and cancer (Peduto et al., 2009; Shields et al., 2010; Link et al., 2011), but only on FRC-like stromal cells. Recently, Peters et al. (2011) found that T_H_17 cells generated *in vitro* and those found in inflamed CNS tissue of mice with EAE express PDPN (**Table 1**). Administration of a PDPN blocking antibody to mice with EAE did not attentuate disease severity, but significantly reduced the number of ectopic germinal centers induced by T_H_17-mediated disease. While the mechanism of PDPN function in T cells is not yet clear, it likely plays an important role in regulating T cell physiology in inflamed tissues.

Podoplanin expression has been observed on some macrophage subsets (**Table 1**). It was first found on F4/80^+^ macrophages in the red pulp of the spleen. These PDPN^+^ macrophages exhibited marked phagocytic potential and elevated numbers in mice following systemic zymosan treatment (Hou et al., 2010). PDPN is also expressed by inflammatory macrophages such as thioglycollate-elicited peritoneal macrophages and LPS-treated RAW264.7 cells (Kerrigan et al., 2012). These studies showed that expression of PDPN by macrophages was sufficient to induce CLEC-2-mediated aggregation of platelets *in vitro*. While the *in vivo* functions of PDPN expression by hematopoietic cells have not been fully elucidated, interesting implications abound given what is known about PDPN function in cancer and autoimmunity.

## PDPN FUNCTIONS IN CANCER

The setting in which PDPN has been most extensively studied is cancer. Given that it is a specific marker of lymphatic vessels, and that increased lymphangiogenesis is often correlated with poor prognosis in cancer patients, the numbers of PDPN^+^ vessels in a tumor is often used as a diagnostic marker (Breiteneder-Geleff et al., 1997; Ji, 2006; Swartz and Lund, 2012). Additionally, PDPN is upregulated on tumor cells themselves in several cancer types, including squamous cell carcinoma of the lung, head, and neck (Kato et al., 2005; Martín-Villar et al., 2005; Schacht et al., 2005; Wicki et al., 2006), malignant mesothelioma (Kimura and Kimura, 2005; Ordóñez, 2005), and brain tumors (Mishima et al., 2006; Shibahara et al., 2006). PDPN is often expressed at the leading invasive edge of tumors and appears to play a role in EMT, invasion, and metastasis (Martín-Villar et al., 2006; Wicki et al., 2006). Interactions between CLEC-2 and PDPN in tumors also likely play a role in tumor progression and metastasis due to platelets interacting with tumor cells (Lowe et al., 2012). However, the exact mechanism of PDPN action in tumor cells is still unclear; in some cases, PDPN expression mediates the downregulation of E-cadherin and promotes EMT (Martín-Villar, 2006), while in others, PDPN expression enhances tumorigenesis and metastasis in the absence of EMT (Wicki et al., 2006). *In vitro* studies have provided compelling evidence that forced expression of PDPN in cells that normally lack this protein results in a more mesenchymal phenotype, actin-rich filopodia, and increased migration and invasion, as discussed above (Martín-Villar, 2005,^2006^; Wicki et al., 2006).

Interestingly, PDPN is also upregulated by cancer-associated fibroblasts (CAFs) in the stroma surrounding various tumors, including adenocarcinomas and colorectal cancers (Kitano et al., 2010). There is a wealth of data on the tumor-promoting effects of CAFs, which has been reviewed elsewhere (Kalluri and Zeisberg, 2006; Gaggioli et al., 2007), but only recently have specific functions for PDPN on CAFs been examined. Generally, the expression of PDPN on CAFs is associated with poor prognosis: for example, one study found that invasive adenocarcinomas in the lung had PDPN^+^ fibroblasts, while non-invasive cases were all negative for PDPN staining (Kawase et al., 2008). Further studies from this group have examined the mechanism by which PDPN enhances the tumor-promoting effects of CAFs. They found that fibroblasts isolated from the vascular adventitia (VAFs) were better at promoting tumor growth than fibroblasts isolated from human lungs. One of the most differentially expressed genes in these cells was PDPN, and knockdown of PDPN in the VAFs abrogated their tumor-promoting effects (Hoshino et al., 2011). Further studies indicated that this activity may be due in part to increased RhoA activity in the PDPN^+^ fibroblasts (Ito et al., 2012).

While these studies illustrate that PDPN expression in CAFs is linked to poor prognosis for patients, it is important to keep in mind that the effect of PDPN^+^ CAFs likely depends on the type of tumor cells and the tissue from which the CAFs originate. In fact, one study of colorectal CAFs found that PDPN expression was correlated with a better prognosis (Yamanashi et al., 2009). Knockdown of PDPN in CAFs resulted in enhanced cancer cell migration in a transwell assay. Furthermore, PDPN expression was seen in stroma surrounding the tumors in many areas except at the invasive front (Yamanashi et al., 2009). Thus, it was postulated that PDPN expressing stroma could act as a physical barrier to tumor cell invasion into surrounding tissues. In fact, this theory has been presented elsewhere and for other mucins (Zimmer et al., 1999). The negative charge of the many sialic acids on these proteins acts to repel other molecules such as complement (Michalek et al., 1988; Meri and Pangburn, 1990) and can affect cell adhesion (Taylor and Drickamer, 2007). Whether these properties play a role in PDPN function has not been definitively examined but it is an attractive hypothesis, given that PDPN is expressed on the apical surface of many cells that have contact with proteinase-rich fluids (i.e., lymph).

While it is clear that PDPN plays an important role in tumor progression and metastasis, more mechanistic studies are needed to fully elucidate the function of this molecule. Furthermore, a genetic dissection of PDPN function in malignant cells versus in the surrounding tumor stroma will significantly advance our understanding of this molecule in cancer.

## CONCLUSION

Emerging studies of PDPN suggest that this molecule plays diverse roles throughout the body. It is involved in the development of the heart, lung, and lymphatic system as well as driving inflammatory diseases and metastasis. The majority of mechanistic data available on the cellular functions of PDPN come from studies of cancer progression and metastasis. Overexpression of PDPN in various cell lines results in increased motility and a mesenchymal phenotype *in vitro* and increased metastasis *in vivo*. These changes occur through the interaction of PDPN with ERM proteins and subsequent modulation of the Rho proteins and actin cytoskeleton. While these studies are indispensable to our understanding of how PDPN functions, it is also critical to examine PDPN in physiological settings, which we have begun to do only recently. Studies of PDPN on LECs and FRCs have indicated that it plays a critical role in mediating interactions with platelets and DCs; however these studies have largely focused on the effects of CLEC-2 engagement of PDPN rather than downstream effects in the PDPN-expressing cell. Furthermore, recent studies of PDPN expression by leukocytes have demonstrated that PDPN expression has intrinsic effects on these cells as well as tumor cells.

There are still many unknowns about PDPN biology that remain to be answered, but there are three pressing questions in the field: (1) What signaling pathways does endogenously-expressed PDPN employ? It is possible that expression of PDPN in leukocytes leads to similar downstream changes as in tumor cells; however, it is likely that PDPN interacts with different molecules and signaling pathways in stromal cells and leukocytes than in malignant cancer cells. (2) What are the effects of CLEC-2 engagement of PDPN? This interaction has been almost exclusively studied with respect to signaling downstream of CLEC-2. However, in nearly every instance where PDPN is expressed, whether by FRCs or cancer cells, there will be CLEC-2^+^ cells in the nearby environment, including DCs or platelets. Given that overexpression of PDPN has striking intrinsic effects on various cells, it stands to reason that there could be some effect on PDPN signaling when it is bound by CLEC-2. (3) What are the consequences of deleting PDPN from cells that endogenously express it? This question has been partially answered by studies of the developing heart, lungs, and lymphatic system, but research has been limited by the lack of a conditional PDPN knockout mouse. Once this tool is generated, we will be able to more closely examine the effects of PDPN in adult animals and in specific tissues or cells. These studies will provide critical insight into whether PDPN is necessary only during embryonic development or into adulthood for proper development and maintenance of organs. Furthermore, we can study how the deletion of PDPN in macrophages or T cells affects disease progression. A better understanding of these open questions will lead to great insights in the diverse fields of development, cellular interactions in the immune system, and cancer progression and metastasis.

## Conflict of Interest Statement

The authors declare that the research was conducted in the absence of any commercial or financial relationships that could be construed as a potential conflict of interest.
